# Mercurius Vivus

**Published:** 1869-12

**Authors:** C. S. Chittenden


### ARTICLE X.

### *Mercurius Vivus.*

By C. S. Chittenden.

Some year and a-half or two years ago, Dr. H. S. Chase, now of the *Missouri Dental Journal*, called the attention of the profession, through one or more of the dental journals, to the efficacy of the Homœopathic preparation of mercury, called mercurius vivus, in the treatment of periostitis. It was a new thing to me, and as I, in common with most of the dentists of the province, was, and am frequently troubled with patients returning and complaining of more or less tenderness about the roots of teeth, after having had the nerves extirpated, and the roots filled, thus indicating that inflammation of the periosteum had supervened, I resolved to test Dr. Chase's prescription. Accordingly I called at one of the Homœopathic Pharmacies and asked for the drug. On being told for what purpose I wished to use it, the person in charge replied, "We have emplowed it for tenderness of the teeth for years, with marked success." I procured two ounces of the third decimal trituration, enough to last long enough to test the thing thoroughly, and waited for the first patient. For the purpose of giving the result of my treatment of this vexatious disease, and of inducing others to try this remedy, I give a short history of a few cases in which I employed the drug.

August 7th, 1868.—Filled the roots and crown of the first left superior molar, for Mrs. E. M.———, aged about 25; strong and healthy. Aug. 10th, Mrs. M———, called to say that she had been suffering for some hours with a dull heavy pain in the tooth which I had filled, but the pain had increased so much that she could bear it no longer. I gave her four doses of the mer. vivus, each dose containing about as much as would lie on a five cent piece, and requested her to take them at intervals of three hours. Aug. 12th, Mrs. M———, called, pain all gone, and tenderness nearly so.

*Case* 2.—Mr. T. C——, called to consult me with regard to the right central and lateral incisors of the lower jaw. On examination I found that the teeth were not decayed at all, but were slightly discolored, and very tender to the touch and had been so for two or three days. I decided at once that the nerve had been injured or destroyed by a blow on the teeth, or a fall, by which they had been loosened, sometime in the man's early life, but he could not recall any accident of the kind. He stated that a few days before, in biting a hard biscuit, he had felt a slight twinge of pain in those teeth, and that the soreness commenced from that date. I resolved to try the mer. vivus in this case, too, as the nerve cavity in these teeth is so small that there would be less fear of trouble from its acting as a reservoir for holding fetid matter arising from the decay of the dead nerve, than is usually found in teeth whose nerves are large. Accordingly, I gave him four powders, and directed him to take them at intervals of four hours. Two days after he reported himself free from pain and the soreness nearly all gone.

*Case* 3.—Miss M———, of Belleville, called about a severe tenderness of a left superior bicuspid, which had been filled a few days before, by Dr. Relyea. The nerve had not been uncovered while being prepared, but the dentine had been exceedingly sensitive. For a week after the filling, the tooth had given no annoyance, but, then Miss M——, began to feel a slightly painful sensation on closing her teeth together which increased in severity till she called on me. I prescribed mer. vivus as in Cases No's. 1 and 2, but she objected that her family were allopathic, and she didn't believe in "sugar pills." However, after my assuring her that the medicine could not injure her she consented to take it, and promised to come back and let me know its effect. Three days after she called and told me that she had been entirely relieved in a few hours after taking the drug.

I might relate a score or two of similar cases but these will suffice to show that this drug may be used with decided benefit under certain circumstances, and I refer to these for the purpose of inducing others to try it. It is to be hoped, however, that no dentist will exercise less *care* in treating teeth whose nerves have been devitalized, than he otherwise would because a remedy has been found which acts most beneficiently when disease supervenes, after the *greatest care* has been taken with such teeth.—*Canada Journal Dental Science.*

